# Erectile dysfunction: Is platelet-rich plasma the new frontier for treatment in patients with erectile dysfunction? A review of the existing evidence

**DOI:** 10.3389/frph.2022.944765

**Published:** 2022-08-16

**Authors:** Eleni Anastasiadis, Razna Ahmed, Abbas Khizar Khoja, Tet Yap

**Affiliations:** ^1^Department of Urology, Croydon University Hospital NHS Trust, London, United Kingdom; ^2^Guy's, King's and St Thomas' School of Medical Education, King's College London, London, United Kingdom; ^3^Department of Urology, Guy's and St Thomas' NHS Foundation Trust, London, United Kingdom

**Keywords:** platelet-rich plasma (PRP), regenerative medicine, erectile dysfunction, male infertility, biotechnology

## Abstract

Erectile dysfunction (ED) is one of the commonest disorders in adult males and affects 12–19% of men of reproductive age. Only few studies have evaluated the impact of ED on men and couples with infertility—these studies report higher rates of ED in this sub-group of men compared to the general population, with the prevalence of ED in men diagnosed with male infertility ranging from 6.7 to 61.6%. Nevertheless, ED is considered a rare cause of male infertility, accounting for about 0.4–5% of all causes of male infertility. ED remains a poorly treated condition globally and current therapies, like oral medication, offer only temporary symptomatic relief and do not influence disease progression—patients are potentially on lifelong treatment, with ED worsening over time. In contrast, regenerative medicine may potentially reverse or halt the progression of ED processes. In this article, we review the evidence for intracavernosal injections of platelet-rich plasma (PRP) in the treatment of ED.

## Introduction

Erectile dysfunction (ED) is one of the commonest disorders in adult males, affecting an estimated one in five (4.3 million men) across the United Kingdom (UK) (1). By 2025, 322 million men worldwide will be affected by ED, with prevalence estimates reported as up to 48% (2–5). This prevalence increases with age, from 5% in men aged 20–39 years to 70% in men aged >70 years (2). There are a multitude of causes of ED, including psychogenic and organic causes which are extensively discussed elsewhere (6, 7). ED manifests due to reduced penile arterial blood flow, nerve and endothelial dysfunction.

Despite this high prevalence and its association with many conditions, ED remains a poorly treated condition globally (8–10). ED imposes a significant quality of life and economic burden on men and their partners. A meta-analysis (*n* = 22,527) revealed that ED increased the risk of depression by 192% (11). Partners of ED patients are significantly impacted due to relationship difficulties and sexual dissatisfaction (12, 13). In addition, men with ED had significantly higher rates of absenteeism and work productivity impairment compared to men without (2).

ED affects 12–19% of men of reproductive age (6). Only a few studies have evaluated the impact of ED on men and couples with infertility—these studies report higher rates of ED in this sub-group of men compared to the general population (6). Possible explanations for this observation may be the psychological disturbances associated with both conditions (anxiety and depression), decreased general health status and the use of medications to treat underlying health conditions (6). The prevalence of ED in men diagnosed with male infertility ranges from 6.7 to 61.6% in large cohort studies (6). Despite this high prevalence of ED in men experiencing infertility, ED is considered a rare cause of male infertility, accounting for about 0.4–5% of all causes of male infertility (6, 7, 14). ED can impair fertility through absent erections, insufficient erection for penetration, and reduced frequency of sexual intercourse (6). Male infertility itself may be the cause of the ED by its negative impacts on sexual, psychological and marital life, including the impact on female sexual function (6).

Current therapies to treat ED include oral PDE5i medications, intracavernosal injection or intraurethral applications of vasodilators (e.g., alprostadil), vacuum erection device, and ultimately if the aforementioned fail, penile prosthesis implant. However, these treatments offer only temporary symptomatic relief and do not influence disease progression—patients are potentially on lifelong treatment, with ED worsening over time. Side effects from oral medications affect more than 16% of men, and more than 50% of men stop using oral tablets due to adverse effects, interactions with other medication, and variability in effect (10). As ED progresses, fibrous tissue replaces smooth muscle in the penis, rendering it inelastic and unresponsive to medication (10). Although penile implants do offer a long-term solution for ED, it is a treatment usually reserved for end stage ED, and does not treat or reverse the underlying pathophysiological mechanisms that result in ED. In contrast, regenerative medicine may potentially reverse or halt the progression of ED processes (15).

Platelet rich plasma (PRP) is an exciting biotechnology that has been shown to stimulate and accelerate bone and soft tissue healing (16). Hematologists used PRP in the 1970's to describe plasma with a platelet count above that of whole blood, which was used as a treatment for patients with thrombocytopaenia (17). A decade later maxillofacial surgeons started to use PRP and currently PRP is used in a multitude of specialities—orthopedics/musculoskeletal injuries, cardiac surgery, plastic surgery, dermatology etc although admittedly with mixed results (17–22). PRP acts on cells to increase their numbers (mitogenesis) and stimulate vascular ingrowth (angiogenesis) and thus promote healing (16). This is because platelets do not only have haemostatic properties, but they also contain an abundance of growth factors (GFs) and cytokines that can affect inflammation, angiogenesis, and cell proliferation (17). These GFs and cytokines are released upon platelet activation—the most important GFs include vascular endothelial GF (VEGF), fibroblast GF (FGF), platelet-derived GF (PDGF), epidermal GF (EGF), hepatocyte GF (HGF), transforming GF beta-1 and beta-2 (TGF-b1/2), insulin-like GF (IGF−1, IGF-2), interleukin 8 and matrix metalloproteinases 2,9 (16, 17). Studies postulate that PRP injections may modify key pathophysiologic mechanisms leading to ED through anti- inflammatory, reparative, neuroprotective and neurotrophic effects (23–26).

In this article, we provide a brief overview of what PRP is, how it is obtained and how it may be administered in ED. We also review the literature and consider the current evidence base for its use and make suggestions for future studies.

## What is PRP and how is it obtained?

PRP is autologous plasma that has a platelet concentration above the concentrations typically found in whole blood—platelet counts in blood can range between 150,000 and 350,000/μL. Platelet concentrations of more than 1,000,000 platelets/μL have been shown to enhance soft tissue healing and thus is the working definition of PRP (16).

PRP is derived from the patient's own blood (autologous) through a process of centrifugation (usually a single or double centrifugation technique) which separates whole blood into its different components depending on their different density gradients—red bloods cells, leucocytes, and platelets/PRP—platelets having the lowest density is the top layer. A small volume of blood is obtained from the patient into a tube that contains an anticoagulant (usually acid citrate dextrose or sodium citrate solution) before centrifugation. The upper-most PRP layer is removed and then administered *via* an intracavernosal injection. The addition of Thrombin and calcium chloride to the PRP can be used to activate the platelets before the injection (to stimulate the release of GFs) however there is currently no consensus on whether this is required for optimal results and with which agent (17). Several CE medical devices are available to produce autologous PRP however they vary in the timings and speed of centrifugation and thus each device may produce different types of PRP depending on their ability to concentrate platelets (17). In addition, devices should aim not to damage the platelets (16).

Currently there is no consensus on the volume/dose of PRP that is required, which technique, and what frequency is required to administer the platelets to achieve maximum results.

## Review of the current evidence

### Animal, pre-clinical studies

Various bench-side studies have demonstrated the value of PRP on the regeneration of nerves and tissues.

Ding et al. wanted to evaluate the effect of PRP on regeneration of cavernous nerves (CN) of Sprague-Dawley rats (23). The reasons for this analysis were because radical prostatectomy for prostate cancer can result in CN injury and as a result, erectile dysfunction. They took 24 rats and divided them into three groups—group 1 underwent dissection of the CN, but nil else (sham group); the second group underwent CN dissection but in addition had a CN crush injury applied bilaterally; the final third group underwent the same procedures as the second group, however PRP was applied to the crush nerves immediately following the crush injury. In this study, PRP was obtained from six age-matched Sprague-Dawley rats. The blood underwent two cycles centrifugation to isolate PRP. The PRP was then “activated” with 10% calcium chloride solution and with bovine thrombin to form a gel for application. The erectile function was assessed in all the rats after 3 months by electrostimulation of the CNs and by measuring the intracavernous pressures (ICP). Following the functional testing, the CNs and mid-shaft penis were collected for staining and histological examination. Results showed that in the PRP treated group, the mean maximal ICP was significantly higher than the injured control group, but it was still less than the sham group. Histological examination demonstrated decreased number of myelinated axons (atrophy), and in the PRP treated group there was a significant increase in the regeneration of well-orientated myelinated axons relative to the control group. This study demonstrated the neurotrophic effect of PRP. The authors concluded that future work was needed to find the optimal PRP dose/preparation, and to identify the primary molecular signaling pathway in the process of nerve repair.

A similar study was repeated by Wu et al. in rats undergoing crush injury to CNs, however in this latter study, the PRP was injected into the corpus cavernosum (CC) (24, 27). In this study rats were divided into three groups—control group with operation only, a group with CN crush injury with PRP injected into the CC, and a third group with CN crush injury with saline only injected into the CC (vehicle-only group). The preparation for the PRP were similar to the methods used in the study by Ding et al. They also measured ICP during electrical stimulation of the CNs, as well as histological analysis (including analysis of tissue from CNs, dorsal penile nerve and CC), after 4 weeks. The results showed a statistically significant recovery of erections after PRP injection compared to the vehicle-only group, with the vehicle-only group showing lower mean maximal ICP compared to the control group. They also found an increased number of myelinated axons showing preservation of nerve axons in the PRP treated group. This group have conducted further studies to further understand the neuro-protective effects of PRP (28, 29).

Liao et al. evaluated the effects of PRP on improving ED in the streptozotocin (STZ)-induced diabetic rats (30, 31). This study was created based on the evidence of the aforementioned effect of PRP on CN regeneration in the rat animal model, and on the pre-existing evidence demonstrating that PRP can prevent the atrophy of corporal smooth muscle cells. Rats who were diabetic and had ED were either given an intracavernous injection of PRP, or the sham group were given injection with the vehicle only. As controls, comparison was made with rats who were not induced with STZ injection to have diabetes and rats who had STZ induced diabetes, but no ED. Four weeks post treatment, the PRP treated group increased all erectile function parameters compared to the sham treated group, but the responses remained less compared to the control groups. Interestingly, histological analysis revealed restoration of damaged corpus cavernosum (CC) after PRP treatment and similarly that nerve damage was reversed with PRP treatment. These findings suggest that PRR can accelerate nerve regeneration and prevent corporal smooth muscle atrophy. Similar studies, with equivalent results have been conducted by Gur et al. and Huang et al. (32, 33).

### Clinical studies

#### Evaluating PRP as monotherapy

Few clinical studies have been done that evaluate the effect of PRP on ED as a monotherapy in humans. Additional studies have been done but this was in combination with other treatment modalities for ED. Key details and summary findings are shown in Table 1, including the methodologies of PRP preparation and technique of intracavernosal injections (ICI).

**Table 1:** Summary of key details of clinical studies evaluating PRP in ED as a monotherapy.

**References**	**Type of study (*n* =** **number included)**	**PRP preparation method**	**Intra-cavernosal injection** **method**	**Primary results**	**Adverse events**	**Limitations**
Poulios et al. (34)	Double-blind, placebo controlled, RCT (*n* = 60)	Autologous platelet separator (Magellan Autologous platelet separator)	- Penile tourniquet for 20 min - 5 mL infused into each corpus cavernosum - Compression bandages after injection	MCID achieved 69% in PRP group vs. 27% in placebo group, *p* < 0.001	None observed	- Small cohort of men - Highly selected, single center study population that may lack external validity
Alkhayal and Lourdes (Conference abstract) (35)	Prospective cohort study (*n* = 61)	Automatic dual spin Magellan Arteriocyte machine.	According to American Cellular Medicine Association protocol (unclear from abstract the exact method used, not clear how many injections each patient received)	Mean IIEF5 score before treatment 12.5 (range 5–20) vs. post treatment mean IIEF5 17 (range 5–24), *p* < 0.001	None reported	- No placebo or control arm - Large number of losses to follow-up introducing risk of bias - Methods not clearly explained, number of injections not disclosed - Variable follow-up - Mean follow-up only 11 weeks
Banno et al. (Conference abstract) (36)	Prospective Cohort study (*n* = 9)	Not disclosed	One injection only, method of injection not disclosed	Average IIEF5 score prior to injection was 15.6 (range 12–20) vs. 19.9 (range 11–27) (*P* = 0.157)	None reported	- Moderate ED patients only - Only nine patients included in study - PRP injection used in combination with standard treatments - Variable follow-up period - Likely follow-up period very short
Matz et al. (37)	Retrospective cohort analysis (*n* = 5 men with ED)	- Centrifuged at 6,000 RPMS for 6 min - Supernatant separated using a proprietary system. - 10% CaCl solution added in a 1:10 ratio (“activated”)	- Intracavernosal injection method not described in detail - Average 2.1 injections	IIEF-5 scores improved by an average of 4.14 points after PRP therapy (baseline IIEF not reported, result included one patient with Peyronie's disease)	- Mild pain (24%)	- Very small number of men with ED - Heterogenous group of men - severity of ED not evaluated - No baseline IIEF scores were reported - No statistical analysis of results
Taş et al. (38)	Prospective cohort analysis (*n* = 31)	- Centrifuged at 2,800 rpm for 8 min. - The plasma layer centrifuged again at 3,500 rpm for 10 min. - Solution containing 1,000e2,000 103/μL PRP was prepared.	- Three intracavernosal injections with 15-day intervals between each injection - Topical anesthetic cream (25 mg lidocaine and 25 mg prilocaine) LA was applied - Clamping (20 min) was performed with Stockmann penis clamp - 3 ml PRP was injected into each corpora cavernosa - The injection sites vary by 1 cm in the mid-penile region	- IIEF5 score improved from 18 to 20 (*P* < 0.001) - 61.29% of men improved	- Bruising in 8.6% of injections - One patient developed 4 mm fibrotic plaque (ventral penis), this was not bothersome	- Evaluated men with metabolic syndrome (thus results may not be generalizable to other causes of ED) - Modest improvement in IIEF
Zaghlou et al. (39)	Prospective cohort study (*n* = 34)	- Centrifuged for 5 min at 1,000 rpm, room temperature. - Manual counting of the platelets on a sample of 10, the platelet count was found to increase by about 8-fold - Plasma layer was again centrifuged for 5 min at 3,000 rpm, room temperature. - The pellet was kept with 1 ml plasma	- Injections once per week for 2 months - 0.5 ml of PRP concentrate in each corpus cavernosum - After PRP injection, direct pressure was applied to the base of the penis for 1–2 min - Massage of the penis was also done for 1–2 min following the injection	- Mean increase of IIEF5 score by 5.5 points (*P* < 0.001) - On multivariable analyses that smoking and baseline IIEF scores independently predicted response to PRP injections (*p* = 0.040 and *p* = 0.023, respectively	- None reported	- Very short term, evaluated men 3 months after starting injections - Small cohort, no control/placebo group - All patients were prescribed PDE5i medications for month after the PRP injections were completed
Geyik (40)	Retrospective cohort (*n* = 184)	- Centrifuged at 3,700 Å~g for 10 min - Supernatant was separated from the remaining blood sample. - Two kits yielded ~12–16 ml of injectable PRP	- LA cream was applied at least 20 min before injection. - Three injections given, 10–14 days apart - ~3–4 ml of PRP was injected in each one of the four regions: one intracavernosal and three subcutaneous areas (both right and left lateral neural lines and dorsal balanic submucosal region	- Increase in mean IIEF score of about nine in both groups - Li-SWT and PRP combination treatment did not show an improvement in IIEF scores compared to Li-SWT alone	- Temporary pain at the site of injections - Mild penile bruising (26%)	- Study assessing combination therapy—men either underwent Li-SWT or Li-SWT and PRP injection - Men were still using PDE5i medication throughout the study - No randomization (patients chose their treatment) - Differences in age and mean hypertension rations between the two groups - No placebo injection
Ruffo et al. (Conference abstract) (41)	Prospective RCT comparing Li-SWT alone (Group 1) vs. LI-SWT plus PRP IC injection (Group 2) (*n* = 100)	Blood samples were centrifuged at varying speeds until separated into three layers—PRP layer used	- PRP injected directly into corpora as a single shot - One injection per week for 6 weeks	- Group 1-mean score increased from 14.6 to 17.3 (*P* < 0.03) - Group 2—mean score increased from 13.7 to 20.2 (*P* < 0.001) - Intergroup analysis of IIEF *p* < 0.001—Combination therapy significantly increased scores compared to monotherapy	- None reported	- Follow-up after 12 and 24 weeks - Total 100 patients allocated 1:1 - Not blinded - No placebo injection given - Abstract only, so information and details on injections etc. limited
Chalyj et al. (42) and Epifanova et al. (43) (abstract/review)	- Cohort study - Study to optimize PRP production (*n* = 75)	- PRP activated with 10% calcium chloride solution - PRP activated with 10% calcium chloride combined with PDE5i medication - Inactivated PRP	No data on how injections given or how many given	- Follow-up from day 28 - Statistically significant results seen in all groups	None reported	- Article in Russian - Results reported in review article by Epifanova et al. (44), report not complete - No baseline data given about the group with regards to severity of ED, and baseline IIEF scores, and characteristics or causes of ED - No placebo group - Variable follow-up period - Follow-up up to max of 6 months
Shin-Mei et al. (45)	Prospective single-arm cohort study (*n* = 30)	- 30 ml blood drawn from each patient - Double spin technique at 500 and 1,500 G for 15 min. - 1–2 ml PRP then obtained	- Three sessions of PRP IC injections 3 weeks apart - One injection of PRP given into each CC (bilateral)	- Assessment were done at baseline and 2 weeks after each injection - Significant improvement of 4.556 IEEF5 score (*P* < 0.001), and 0.72 points in EHS (*P* < 0.001) - Overall, 82.8% participants found overall improved erectile function.	- No significant adverse events reported - Small induration at injection site	- No placebo or control groups for comparison - Single institution - Short follow-up—assessment was done 2 weeks after each injection session and longer-term outcomes not assessed - PDE5i medication and testosterone replacement therapy was allowed

Only one randomized, double blind, placebo-controlled study has been conducted and published to date evaluating the effects of PRP in men with mild and moderate ED (34). This was conducted by Poulios et al. and published in 2021, and is the highest quality study to date of PRP injections in ED. They randomized 60 men to receive either 10 ml PRP or placebo (*n* = 30 in each arm) ICI (after a washout period of 1 month). The preparation of the PRP included the blood sampling in a 60 ml syringe containing 8 ml of anticoagulant. The blood samples were then processed by the FDA-approved autologous platelet separator (Magellan Autologous platelet separator) to yield about 10 ml of PRP. This system was used as it is thought to yield high quality PRP. A penile tourniquet was applied around the base of the penis and about 5 ml of PRP ICI was given. The tourniquet was released after 20 min. Patients were evaluated at 1, 3, and 6 months after the injections. The primary outcome was the proportion of men who achieved the “minimal clinically important difference (MCID)” in the International Index of Erectile Function (IIEF5) from baseline to 6 months, as well as the safety of the injections. They found that at 6 months, 69% of men in the PRP group vs. 27% of the men in the placebo group achieved MCID (risk difference of 42%, *P* < 0.001). Statistically significant results were also achieved at the 1- and 3-month follow-up period. This study was a single-center study and therefore lacked generalizability. It was also a small study that had very strict inclusion criteria (for example, it only included heterosexual males in a stable relationship). In addition, the study could not be extrapolated to other PRP separation systems.

Other non-randomized studies have been conducted—some of which have only been published in conference abstract form.

Alkhayal et al. reported on their findings of 267 men who had organic, mild to moderate erectile dysfunction who received the PRP injections (in a conference abstract) (35). They also evaluated using the IIEF5 questionnaire and assessed men at least 6 weeks after treatment. However, the group only had full data on 61 patients and from the abstract it is not clear what happened to the rest of the men and why they may have dropped out. Mean follow-up was for 11 weeks only. Mean IIEF5 score before treatment was 12.5 (range 5–20) (considered mild-to-moderate ED) and post treatment mean IIEF5 score was 17 (range 5–24) (mild ED), *P* < 0.001. There were no adverse events reported. Although this study does show a positive result with the use of PRP, there is no placebo-controlled group in the study. In addition, there was a very large number of dropouts or missing data, which brings into question the possibility of systematic bias in the results and would also flag up concerns with regards to adequate reporting of adverse effects. This was single institutional study and thus may lack external validity. The mean follow-up period was very short, and the longer-term effects could not be evaluated. Finally, the difference in mean IIEF5 scores before and after treatment show an improvement of 4.5 points on the IIEF5 questionnaire (from mild-to-moderate ED at baseline to mild ED post treatment) and it is arguable whether this represents a clinically significant improvement in erectile function.

Similarly, Banno et al. reported on a small cohort of men within their institution who received PRP injection (one injection only) in combination with standard of care treatment (medication and vacuum therapy) for their ED (36). The effects of PRP were assessed at least 4 weeks post injection. Only nine patients were included in the study. Average IIEF5 score prior to injection was 15.6 (range 12–20) and at least 4 weeks post injection the score was 19.9 (range 11–27). The difference in scores did not achieve statistical significance, however the sample size was probably too small to observe an effect. Again, the follow-up period in this study was limited. No adverse events were noted.

Matz et al. reported on a mixed cohort of patients with urological conditions who received PRP in a retrospective cohort analysis study (37). This study evaluated the use of PRP in men with ED, Peyronie's Disease and stress urinary incontinence. They evaluated only 5 men with ED who received on average 2.1 injections during the study period. In this study, the autologous PRP was activated by mixing the PRP with a calcium chloride solution and injected into the patient within 10 min of final preparation. The thought behind this was to create a mixture that would have longer local retention within the tissues and to avoid early washout. There was an improvement of IIEF5 score of 4.14 points. Of course, this was a very small cohort of men, retrospective in nature and contained no placebo-controlled group for comparison. In addition, the baseline IIEF5 scores were not reported in the paper, and it is not clear whether the men with ED included in the study had mild, moderate, or severe ED. The latter point is important, as it might help to identify those men most likely to benefit from PRP injections, and is an important oversight. However, consistent with previously mentioned studies and reports, there were no adverse events noted in men receiving PRP injections in the ED group.

More recently Taş et al. published their findings (38). This prospective cohort analysis evaluating the effect of PRP in 31 patients with ED (more specifically, in men with metabolic syndrome aged between 42 and 70 years of age). They gave three ICI with 15-day intervals between each injection. Patients were followed up prospectively and re-evaluated at 1, 3, and 6 months after the injections. The results showed that the mean IIEF5 score improved from 18 to 20 (*P* < 0.001), however despite this improvement the score remained within the mild IIEF5 ED classification system (score of 17–21). They reported the mild side effect of bruising in 8/93 injections administered at the injection site. One patient developed a 4 mm diameter fibrotic plaque on the ventral side of the penile shaft. This did not cause the patient any pain, curvature or shortening of the penis and was not noticed by the patient. In total, 19 patients (61.29%) improved after 6 months. Again, this was a small cohort of men, with no placebo group for comparison. The men included in this study had metabolic syndrome, and thus the result may not be extrapolated to men who have different causes of ED and to men without metabolic syndrome. In addition, the improvement in IIEF5 scores is modest. Nevertheless, it does provide useful data on some the adverse events/side effects that may be expected especially from multiple injections.

Zaghlou et al. examined the effects of smoking status and baseline IIEF5 in the response to PRP (39). The study was aimed to evaluate the effect of PRP ICI in men with ED whose ED was not responsive to PDE5i oral medication (including daily and on-demand regimes). They prospectively studied a cohort of 34 men with ED. IIEF5 was documented for each patient before and 1 month after completion of PRP injections. Penile hemodynamics was also assessed with a pharmacologically induced erection duplex penile USS before and 1 month after completion of PRP injections. PRP injections were given once per week for 2 months. In addition, all patients were prescribed PDE5i medications for a month after the PRP injections were completed. PRP preparation in this study did not include activation of the PRP. In this study, after PRP injection, direct pressure was applied to the base of the penis for 1–2 min to prevent rapid washout of the PRP, and massage of the penis was also done for 1–2 min following the injection to distribute the PRP throughout the penile tissues. Results showed a significant increase in IIEF5 score with a mean increase of 5.5 points. They also found on multivariable analyses that smoking and baseline IIEF5 scores independently predicted response to PRP injections (*p* = 0.040 and *p* = 0.023, respectively). Non-smokers were more likely to be responsive to PRP ICI. There were no differences in the penile USS haemodynamic parameters seen after the PRP injections compared to the USS prior to the injections. In addition, 14 patients in the study who were previously non-responders to PDE5i medications showed an improvement in response to taking these medications. Again, this was a cohort analysis of men with no placebo or control group. In addition, it is difficult to understand the direct effect of PRP alone in this cohort study, given that the men were given PDE5i medication for 1 month following the injections. It is also not clear if the IIEF5 scores reported, are scores based on men's response with the use of PDE5i medications or their ED without the use of these medications. The findings that smoking and IIEF5 scores might affect the response of PRP injections is indeed interesting and worthy of further exploration, and this may prove to be of clinical significance in future guidelines if PRP is accepted as treatment for ED. There were no reported side effects in this study population. This study only evaluated the short-term effects of the PRP injections, and no longer-term data was presented.

Chalyj et al. and Epifanova et al. assessed the safety and effectiveness of PRP in men with ED (42, 46). The article is in Russian, with an English Abstract (42, 46). A report of the main study findings is provided in a review article by Epifanova et al. The study was a prospective cohort study which attempted to evaluate the optimal PRP preparation method. They created the following PRP preparations for ICI: (1) PRP activated with 10% calcium chloride solution (30 patients received this ICI); (2) PRP activated with 10% calcium chloride combined with PDE5i medication (30 in this group); (3) Inactivated PRP (15 patients received this IC injection). Follow-up period was from 28 days to 6 months. In group 1, a statistically significant increase in peak systolic velocity (PSV) (*P* = 0.005) and resistance index (RI) (*P* = 0.001), as well as in IIEF5 score (*P* > 0.046), was observed. In group 2, PSV (*P* = 0.028) and RI (*P* = 0.129) values, as well as IIEF5 score (*P* = 0.046) improved. In group 3, a statistically significant difference was found in IIEF5 score (*P* < 0.05) as well as in PSV and RI (*P* > 0.05) values (46). All groups showed significant improvement in IIEF5 scores over the period, however the changes in penile doppler ultrasound scan parameters were variable between the groups. The authors concluded that PRP contains the amount of growth factors required for therapeutic effects. No adverse events were noted. Limitations of the study include the absence of a placebo or standard treatment group.

Shin-Mei et al. recently published their short-term data of IC PRP injections in a prospective cohort study involving 30 men (45). Mean ago of the study participants was 54.93 years (±8.31 years) with a mean duration of ED of more than 24 months. Men started on PDE5i medications and testosterone replacement therapy were allowed to continue during the study period. Erectile function was assessed using the IIEF5 score and Erection Hardness Score (EHS) among other scores—these scores were taken at baseline and then 2 weeks after every PRP IC injection. Participants received three sessions of PRP injection 3 weeks apart. Each IC injection was given by a single urologist bilaterally. Overall, there was an increase of mean IIEF5 score from 12.034 (±5.10) at the start of the study to 16.59 (+/is 5.5) (*P* < 0.001). Moderate to severe ED was recorded in 57% of patients at the start of the study, this decreased to 20% after treatment. No haematoma, infection or pain was reported. Two participants reported induration at the site of the first injection, with no associated functional impairment. This study is limited by the short-term follow-up data and the small study number.

#### Evaluating PRP as combination therapy

Geyik studied the efficacy of low-intensity shock wave therapy (Li-SWT) alone and in combination with PRP in patients with ED (40). This was retrospective cohort study evaluating the interventions in men with ED not responding to PDE5i medication. The men either underwent Li-SWT or Li-SWT and PRP injection, it should be noted that all men were using PDE5i medication before and during the study period. The 93 patients received the former treatment, and 91 patients received the combination treatment. All patients had one course of Li-SWT, which consisted of 5 applications about 7 days apart. Each application included 1,800 shockwaves to the distal penile shaft and 1,800 to the perineal corpus cavernosum. PRP injections were done with autologous PRP. A total of three injections were given, each one being 10–14 days apart. PRP injections were given in one of each four areas—one intracavernosal, and three subcutaneous areas (right and left lateral neural lines and dorsal balanic submucosal region). IIEF5 was measured at baseline and 6 months following the treatments. Patients continued to use PDE5i medications throughout this study period. Side effects reported after the injection included temporary pain at the site of injections and in 26% of men there was mild penile bruising. The results showed improvement in both groups with respect to mean IIEF scores, with an increase in mean IIEF score of about nine in both groups. In conclusion, Li-SWT and PRP combination treatment was not shown to be superior compared to Li-SWT treatment alone (i.e., no difference between the two groups). Limitations of the study include the fact that the men were not randomized (men chose which intervention they would receive) which could create a systematic bias. The mean age and hypertension ratio was higher in the Li-SWT group—these are important risk factors in ED and thus may have artificially biased the results. And, finally, the study allowed men to continue to use PDE5i medication throughout the study, which may have biased any results toward the null hypothesis of no difference.

Ruffo et al. assessed PRP IC injections plus Li-SWT for ED (abstract only) in a prospective randomized trial assessing Li-SWT alone (two weekly sessions) for 6 weeks or Li-SWT (two weekly sessions) plus PRP IC injection (once per week for 6 weeks) (41, 47). They showed that IIEF scores increased significantly in both groups, with Group 1 showing IIEF mean score increased from 14.6 to 17.3 (*P* < 0.03) and in Group 2 mean IIEF score increased from 13.7 to 20.2 (*P* < 0.001). This significant increase was sustained in Group 2 after 24 weeks of follow-up. These results (unlike the study by Geyik mentioned previously) shows a positive response with the inclusion of PRP to the Li-SWT. The study by Ruffo et al. was randomized (allowing for equivalent distribution between the treatment groups of key characteristics) thereby reducing the risk of bias in the results. They also did not allow patients the use of PDE5i medications. However, the study was reported as a conference abstract and publication of full study details is awaited in a peer-reviewed journal.

A further study reported by Zasieda evaluated the combined use of PRP and Low-Intensity pulsed ultrasound (LIPUS) in the treatment of men with ED in a conference abstract publication (48). They evaluated men with vascular ED (arterial and veno-occlusive) in a prospective clinical study of 64 men with moderate and mild ED (according to IIEF5 scores). The study arm underwent six sessions of PRP ICI (1 ml PRP injection given with one session done weekly) and 12 sessions of LIPUS (two sessions weekly). The control group just underwent the equivalent penile LIPUS sessions. After 12 weeks, IIEF5 score improvement was found in 27 vs. 20 patients in treatment group vs. control group (*P* = 0.047) with no significant side effects observed. The findings in this study have been reported in an abstract, and more details are awaited in further publications (especially with regards to the clinical significance of IIEF score improvements).

Other studies have been done evaluating the combination therapy of Li-SWT and PRP (as well as other combination treatments), however some of these studies lack control groups and thus cannot be used to evaluate the effects of PRP alone in ED (43, 44, 49, 50). It should be noted that Li-SWT has not been proven unequivocally to be of benefit in ED, with data from prospective randomized trials showing conflicting results (7). Studies evaluating combination therapy of Li-SWT and PRP ICI should thus be considered cautiously, especially when no placebo arm is included in the study.

#### Ongoing/future trials

There are several registered clinical trials evaluating PRP in ED.

One such trial, a Phase 2, triple blinded, randomized controlled trial is currently recruiting in a single institution, Puerta de Hierro University Hospital, Madrid (51). They are recruiting men between 40 and 75 years of age and plan to assess for the primary outcome at 28 weeks. This trial is evaluating men with more severe forms of ED (IIEF score between 5 and 16), which is an important consideration, as the effect of PRP may not be as pronounced in men with more severe baseline ED, and thus the results from such a trial may not necessarily be extrapolated to men with milder ED (39). The control group will receive Platelet-Poor-Plasma (PPP), and thus the trial arguably lacks a true placebo group. The study population will remain small at about 50 men.

A more promising RCT is currently recruiting in University of Nebraska (52). This is single masked, placebo-controlled, Phase 3 RCT. The men included in the study are 50–80 years of age. The trial will aim to recruit 179 trial participants. They will follow-up men at 1, 3, and 6 months. This trial will not be fully blinded, and it appears that they are recruiting from a single institution, which may limit generalizability of the results. The PRP injection in this trial will be a single injection in the penis. The outcomes will include the rate of change of IIEF. Men included in the trial will have moderate to mild ED. The only exclusion criteria is previous treatment for prostate cancer—this may create a heterogenous group of men with ED, which may mask the effects of PRP and bias the results toward the null hypothesis.

A further RCT has been registered at the University of Miami, USA, and is currently recruiting (53). This is a randomized, double-blinded, placebo controlled, multicenter phase 2 trial (although it is not clear which additional centers are involved or how many). The target recruitment number is 80. The inclusion criteria include men between 30 and 75 years of age with moderate to mild ED (IIEF scores 11–25). Their inclusion criteria are stringent—for example, men are required to be in stable heterosexual relationships, and to agree to attempt sexual intercourse at least four times per month. The primary outcome will be assessed at 9 weeks of follow-up, although the follow-up period appears to extend to 24–29 weeks for secondary outcomes. Men will receive two sessions of penile injections 1 month apart. The placebo injection would be saline. Their primary outcome will report the percentage of men achieving MCID in IIEF5 scores.

Further clinical trials have been registered, but these trials either do not assess the effect of PRP treatment alone in men with ED (54, 55), or they are not randomized clinical trials with a comparator treatment group (i.e., small cohort, pilot, phase 1 studies) (56).

## Discussion and summary of clinical studies

In summary, only one randomized, placebo-controlled trial has been published to date regarding the use of PRP intracavernosal injections as a monotherapy for ED. This study has shown an impressive response in erections in a highly selected cohort of men and offers an exciting new treatment for ED, a condition that to date, has had no treatment that may reverse the dysfunction. However, despite being the best study to date, the follow-up of these men was only until 6 months post PRP injection and further longer-term follow-up data is required to understand the longer-term impact of the PRP injections on erectile function.

Multiple cohort studies have been done in evaluating PRP ICI in ED. The following limitations with cohort studies remain:

1) The studies are often done in a heterogenous group of men with regards to the causes of their ED, and with regards to the severity of ED. This data is important in identifying men most likely to benefit from PRP treatment.
2) The cohort studies lack a control group for comparison.
3) There is a lack of agreement and standardization in the optimal way to prepare PRP and what the optimal PRP solution should look like.
4) There is also no agreed protocol on how to administer the ICI, with variations in how this was achieved in various studies.
5) Follow-up periods in the studies are often variable and often a very short interval after the PRP injections.

Despite their limitations, the cohort studies published to date have shown a tendency for PRP to improve erectile function and to improve outcomes in men with ED and the results have been encouraging. These studies add to the data on possible side effects and adverse events that may occur, and as such have established the treatment as safe with minimal side effects, and demonstrate high tolerance in men receiving the injections (with men often receiving multiple injections). In addition, some studies have identified various factors that may be predictive of response to PRP injections, providing further insights on ways to optimize treatment for ED. Further, larger scale, multi-center studies are required that are also inclusive of a more diverse population of men.

## Author contributions

TY conceived the idea to undertake the review. TY, EA, RA, and AK conducted the literature review and review of the evidence. EA and TY drafted the manuscript. All authors contributed to the final manuscript.

## Conflict of interest

The authors declare that the research was conducted in the absence of any commercial or financial relationships that could be construed as a potential conflict of interest.

## Publisher's note

All claims expressed in this article are solely those of the authors and do not necessarily represent those of their affiliated organizations, or those of the publisher, the editors and the reviewers. Any product that may be evaluated in this article, or claim that may be made by its manufacturer, is not guaranteed or endorsed by the publisher.
